# Surgical Gesture Recognition in Laparoscopic Tasks Based on the Transformer Network and Self-Supervised Learning

**DOI:** 10.3390/bioengineering9120737

**Published:** 2022-11-29

**Authors:** Athanasios Gazis, Pantelis Karaiskos, Constantinos Loukas

**Affiliations:** Laboratory of Medical Physics, Medical School, National and Kapodistrian University of Athens, 115 27 Athens, Greece

**Keywords:** artificial intelligence, machine learning, CNN, transformer, laparoscopic surgery, surgical action recognition, self-supervision

## Abstract

In this study, we propose a deep learning framework and a self-supervision scheme for video-based surgical gesture recognition. The proposed framework is modular. First, a 3D convolutional network extracts feature vectors from video clips for encoding spatial and short-term temporal features. Second, the feature vectors are fed into a transformer network for capturing long-term temporal dependencies. Two main models are proposed, based on the backbone framework: C3DTrans (supervised) and SSC3DTrans (self-supervised). The dataset consisted of 80 videos from two basic laparoscopic tasks: peg transfer (PT) and knot tying (KT). To examine the potential of self-supervision, the models were trained on 60% and 100% of the annotated dataset. In addition, the best-performing model was evaluated on the JIGSAWS robotic surgery dataset. The best model (C3DTrans) achieves an accuracy of 88.0%, a 95.2% clip level, and 97.5% and 97.9% (gesture level), for PT and KT, respectively. The SSC3DTrans performed similar to C3DTrans when training on 60% of the annotated dataset (about 84% and 93% clip-level accuracies for PT and KT, respectively). The performance of C3DTrans on JIGSAWS was close to 76% accuracy, which was similar to or higher than prior techniques based on a single video stream, no additional video training, and online processing.

## 1. Introduction

Surgical action recognition plays an important role in modern surgical training [1,2]. Specifically, the automatic detection of surgical gestures is of paramount importance for skills assessment and the development of automated training frameworks. Recent advances mainly focus on robotic actions performed in a simulated environment using video or/and tool kinematic data [3], based on the popular JHU-ISI gesture skill assessment working set (JIGSAWS) [4]. Early studies employed the robotic tool kinematics, potentially due to the lower dimensionality involved, with promising results on gesture classification [3,5]. However, using the tool kinematics somewhat limits the application of the employed techniques to robotic surgery, where such data are readily accessible. In this study, we focus on the challenging issue of online recognition of surgical gestures based solely on the video acquired from the endoscopic camera, given that such a technique would also apply to laparoscopic surgery where tool kinematics are typically absent.

Prior work on video-based surgical gesture recognition mainly falls into two broad categories: probabilistic graphical models and recently deep learning (DL) [3]. With respect to the first category, hidden Markov models (HMMs) have been employed extensively in the past [5,6]. Although HMMs employ interpretable features, they focus on a limited number of frames and, thus, the model ignores long-term temporal dependencies. Other popular machine learning methods (e.g., support vector machines, conditional random fields, etc.) assemble multiple heterogeneous handcrafted features (e.g., color, motion) to localize and classify surgical actions [7]. These techniques offered promising performance and explainability, but they leave open the possibility of missing important latent features during the feature extraction process.

The majority of the DL techniques rely on convolutional neural networks (CNNs) [8,9]. A variant of the CNNs is the temporal convolutional network (TCN), which is able to segment and detect actions by hierarchically convolving, pooling, and upsampling input spatial features using 1D convolution and deconvolution [10,11]. This technique showed promising results in handling long-term temporal sequences, though the TCN architecture captures dependencies among local neighbors, thus missing the ability for longer temporal dependencies. Another approach is based on the use of 3DCNNs, which are able, unlike their 2D counterparts, to capture both spatial and temporal features [12,13]. Although this property offers a relative benefit compared to 2D convolutions, 3DCNNs may prove limited in capturing long-term dependencies. In addition, they are resource intensive and potentially impractical for longer time scales. A more recent approach by Zhang et al. [14] employs 2DCNNs, which capture spatial features, and 1DCNNs along with an attention mechanism to capture both short and long-temporal features. This technique proved to be highly successful for robotic gesture detection and segmentation, but it required the whole surgical video as input, thus limiting its application to offline gesture detection. In addition to standard supervised DL, video-based unsupervised techniques have also been employed recently [15,16], but the reported accuracy is significantly lower (about 57% accuracy/silhouette score compared to about 70–90% accuracy reported by offline/online supervised techniques [3]).

As described before, most research on surgical gesture recognition is evaluated on the JIGSAWS benchmark dataset that provides annotated video and kinematic data for three basic robotic surgery (RS) tasks [17]. However, the tools and maneuvers in RS are substantially different from those involved in laparoscopic surgery (LS), where the tools and the camera are controlled directly by the surgeon.

To this end, in this paper, we propose a deep learning approach for laparoscopic gesture recognition based on the transformer architecture and 3DCNNs. To the best of our knowledge, the transformer combined with 3DCNNs or/and a self-supervised model has not been proposed in the literature for surgical gesture recognition. The majority of the proposed video-based techniques employ 2DCNNs (or holistic features) combined with a temporal model (e.g., 1DCNN, LSTM, TCN, etc.), 3DCNNs alone, Markov models with CNNs, and graphical models (CRF, LDS, etc.) [3]. The proposed framework focuses on both fine-grained actions and longer dependencies while retaining the ability for online recognition. The 3DCNN aims to capture short-term spatial features, which subsequently feed the transformer to capture long-term dependencies from longer segments of the video stream. In addition, we investigate a self-supervised training scheme aiming to reduce the necessary annotated data volume. Specifically, we develop an encoder–decoder model for next-frame prediction, aiming to extract potentially useful features that can serve as input to the supervised model (transformer). Hence, extraction of short-term features is completely unsupervised, whereas the learning of long-term dependencies is supervised. This ‘mixed’ type of training proves to be promising when annotated data are limited. Our work is also the first to examine video-based gesture recognition in laparoscopic tasks, which are substantially different from robotic tasks. For this purpose, we developed a laparoscopic video dataset with annotated data for elementary gestures. In addition, the best-performing model was tested on the popular JIGSAWS dataset to further examine its ability for robotic gesture recognition. In summary, our contributions are as follows:

- We propose a novel framework (C3DTrans), based on the transformer architecture and 3DCNNs, for online video-based recognition of surgical gestures.
- We include in the overall pipeline a self-supervised training scheme to investigate its performance in cases where the annotated data volume is limited.
- We develop a video dataset of basic laparoscopic training tasks along with annotated data of the elementary gestures performed.
- In addition to the laparoscopic dataset, the proposed model was tested and compared with other techniques on JIGSAWS for online recognition of robotic gestures.

## 2. Materials and Methods

### 2.1. Task Description

The dataset included videos with training sessions (trials) of two basic laparoscopic tasks: peg transfer (PT) and knot tying (KT). Each task was performed 2–3 times by a group of 15 surgical trainees (residents in post-graduate years 1–3). The mean duration of each trial was 94.3 (PT) and 46.3 (KT) seconds. Overall the dataset consisted of 40 trials for each surgical task.

**Figure 1:**
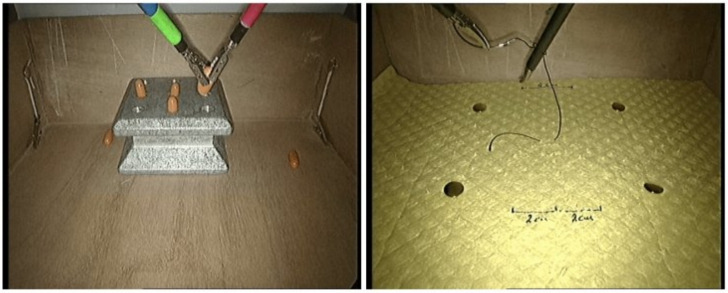
Sample frames extracted from the videos of the surgical tasks (left: PT, right: KT).

Both tasks were performed on a box trainer using standard laparoscopic equipment (rigid endoscope connected to a laparoscopic tower equipped with a DVD recorder). The PT task required the placement of four cylindrical pegs onto a pegboard (two with the left and the right tools, respectively). Each of the first two pegs had to be placed directly on the pegboard, whereas the next two pegs had first to be transferred to the other tool. The second task (KT) was required to perform a single loop knot using a suture with a needle attached at one end. The suture was pre-driven through a tissue pad. The trainee had first to pick up and orient the needle with one tool, then make a C loop and finally reach and pull the free end of the suture with the other tool. Figure 1 shows sample video frames for each task. More details may be found in our prior work [18].

### 2.2. Video Dataset

The video of each trial was recorded at 25 frames per second (fps) and the frame resolution was 720 × 576 (Width × Height). The videos were sub-sampled at 5 fps and rescaled to 224 × 224 (W × H) to fit the input shape of the employed models. In addition, each video was decomposed into smaller video segments, hereafter referred to as *clips* and *volumes*. First, the clips were extracted from the sub-sampled video with a step size = 3 frames and the length of each clip was $T_{L}=15$ frames. Subsequently, the clips were decomposed into volumes with a step size = 1 frame. The length of each volume was $T_{S}=8$ frames, so each clip included $N_{S}=8$ volumes. Compared to the clip, the volume describes a lower granularity level of the surgical task. As described in the following sections, the volumes and the clips serve as input to different modules of the proposed CNN architecture.

### 2.3. Surgical Gestures

The idea of surgical gesture decomposition originates from the language of surgery concept [4], where a surgical task consists of sequential elementary activities (surgemes). In this study, we defined a vocabulary of nine elements to describe the surgical gestures involved in the performance of the two tasks: five gestures for PT and another four gestures for KT (Table 1). This gesture decomposition was proposed in [18] and a similar definition of activities was utilized in [19]. The videos were annotated using Anvil 6.0 [20].

Based on the description of each task, an ideal performance required the following sequence of gestures:

- PT: RP(L) → PP(L) → RP(R) → PP(R) → RP(L) → TP → PP(R) → RP(R) → TP → PP(L)
- KT: RN → ON → CL → PS

A statistical overview of the employed gesture dataset is presented in Table 2. For each gesture, the extracted number of volumes was $N_{S}$ times the number of clips.

### 2.4. Network Architecture

The proposed network architecture is based on two main modules: a short-term (ST) module that captures short-term spatiotemporal features and a long-term (LT) module that captures long-range temporal dependencies. The ST module takes as input a video volume of $T_{S}$ frames and outputs a deep feature descriptor. In this work, we examined two alternative models for the ST module: one based on self-supervised learning (*ST Model 1*) and another one based on standard supervised learning (*ST Model 2*). As described in the next section, the first model can be employed when the available training set of gestures is partially annotated, whereas the second model employs a fully annotated training set.

**Figure 2:**
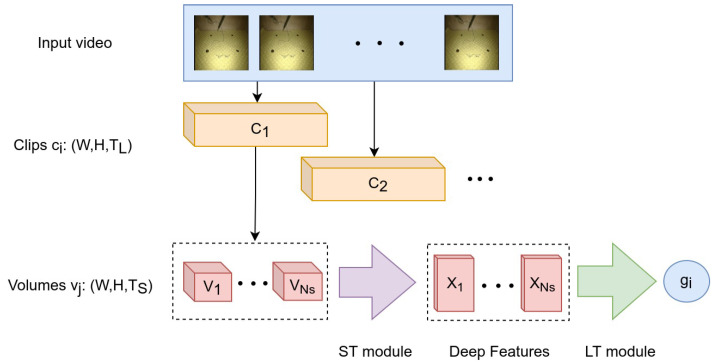
Overview of the proposed architecture using a two-level video decomposition approach. From each clip $c_{i}$ we extract $N_{S}$ volumes $v_{j}$. The volumes are fed into the ST module providing a sequence of $N_{S}$ deep features $X_{i}$, which are then fed into the LT module in order to predict the gesture class $g_{i}$ for the current clip $c_{i}$.

The LT module takes as input a video clip from which a sequence of feature descriptors are extracted using the ST module. Essentially, the descriptors are extracted from an equal number of overlapping volumes inside the clip, as described earlier. Hence, the LT module takes as input a series of descriptors concatenated along the temporal dimension and outputs a prediction about the current gesture performed at the *clip level*. Consequently, our approach does not utilize information about the video frames prior to or after the current clip, thus making the gesture prediction a particularly challenging task. Figure 2 provides a graphical representation of the video decomposition approach and its relation to the ST and LT modules.

#### 2.4.1. ST Model 1: Self-Supervised Learning

The first model is based on self-supervised learning using an encoder–decoder scheme for spatiotemporal feature extraction. Most state-of-the-art CNNs for video gesture recognition rely on supervised learning, demanding a significant effort to annotate the gestures performed during the task [3]. The main motive behind our method was to minimize the annotation effort by employing a larger set of unlabeled data for feature extraction and a smaller set of labeled data for supervised learning (LT module). In addition, in this study, the labeled data do not necessarily need to correspond to the entire length of a gesture, but to short segments. Hence, from each annotated segment a series of clips with the same label is extracted automatically.

Compared to other methods employing static frames [21], here we employ a 3DCNN based on the inflated Inception3D (I3D) architecture [22]. The aim of the ST module is to capture spatiotemporal features related to fine-grained movements performed during the task. The I3D model was based upon the successful Inception architecture by ‘inflating’ the 2D convolutional layers to a new axis that represents the temporal dimension. The original I3D considers two input sources: optical flow and RGB. In this work, the I3D was pretrained on the Kinetics dataset using as input modality only the RGB (https://github.com/dlpbc/keras-kinetics-i3d. Accessed on 20 September 2022).

The self-supervision procedure is based on the idea of network training on a pretext task [23], defined as a self-supervised learning problem where the required labels are automatically generated from the input data. In this work, the pretext task was defined as *next-frame prediction*. Hence, given as input a single volume $v_{j}$, the goal was to predict the next frame in the sequence. The hypothesis that led to this choice was that in order to reconstruct the next frame, the network must be able to create meaningful representations of the input sequence.

**Figure 3:**
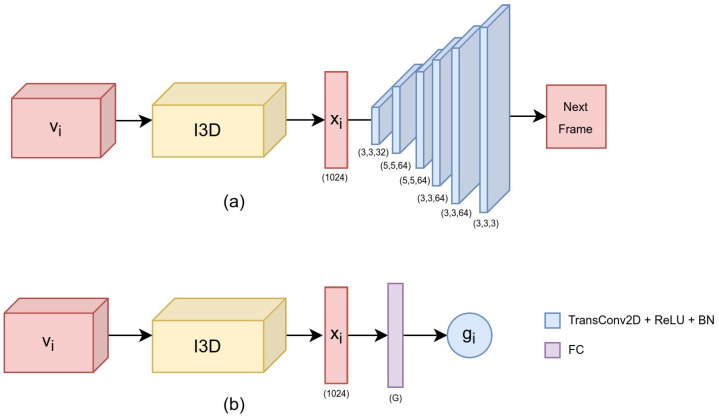
The two ST module architectures employed: (**a**) ST Model 1 and (**b**) ST Model 2.

The input to the ST module is a video volume $v_{i}$ of shape $\left(W,H,T_{S}\right)$. In particular, the volume $v_{j}$ is fed to the I3D network, which acts as an encoder and maps the volume to a feature vector $X_{j}\in R^{1024}$ (also called *embedding representation*). The feature vector is then fed into the decoder, which is composed of 6 consecutive transpose 2D convolutional layers (TransConv2D). Each TransConv2D, except the last one, uses a ReLU non-linearity followed by a Batch Normalization layer (BN). The last TransConv2D uses sigmoid non-linearity and the shape of the output (next frame) is the same as the spatial dimensions of the input volume (W, H). Figure 3a shows an overview of the ST Model 1. The I3D model acts as the encoder, whereas the transposed convolution layers act as the decoder.

#### 2.4.2. ST Model 2: Standard Supervised Learning

For the second model (ST Model 2), the decoder of the previous architecture is replaced by a dense layer with softmax non-linearity (Figure 3b). Similar to the previous model, the I3D Network is fed with a video volume $v_{i}$ of shape $\left(W,H,T_{S}\right)$, which is mapped to a deep feature $X_{i}\in R^{1024}$. However, the feature vector is now followed by a fully-connected layer (FC), with softmax non-linearity, and outputs a vector $Y_{i}\in {\left[0,1\right]}^{G}$, where *G* denotes the number of gestures in the particular task. Consequently, the gesture label $g_{i}$ for the input volume $v_{i}$ is calculated as:

(1) $$g_{i}=argmax\left(Y_{i}\right)$$

#### 2.4.3. LT Module

The LT module was based on the transformer architecture, which was first proposed by Vaswani et al. [24] for natural language processing (NLP), and soon it found applications in the computer vision domain for image recognition [25]. The transformer architecture has recently been applied in surgery for tool detection [26] and phase recognition in laparoscopic procedures [27].

**Figure 4:**
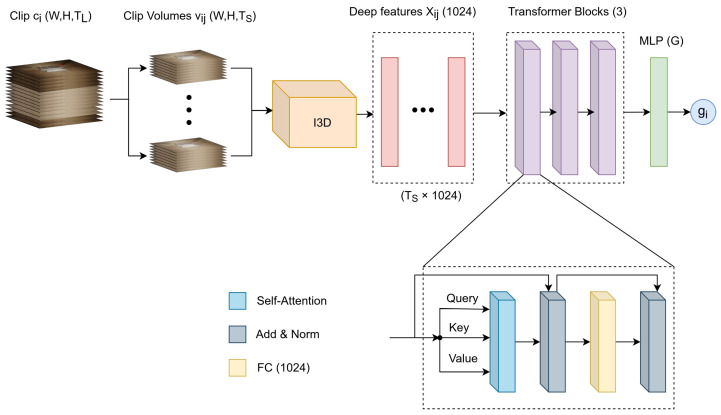
Overview of the transformer architecture.

The transformer employs an attention mechanism [28] to represent relations in time sequences, with the main advantage over other techniques, such as LSTMs being the ability to capture better long-term dependencies. In a nutshell, the transformer architecture employs a variation of attention called scaled dot-product attention. Given a set of a query (*Q*), a value (*V*) and a key (*K*) the attention (*A*) is calculated as:

(2) $$A=Softmax(\frac{QK^{T}}{\sqrt{d_{k}}})V$$

where $d_{k}$ is the dimension of *K*. In this work, we employed scaled dot-product *self-attention*, where the inputs for the *Q*, *K*, and *V* representations are derived from the same input embeddings ($X_{i}$). In particular, the LT module consists of three sequential transformer blocks (TBs). Each TB consists of a self-attention layer, two-layer normalization layers (LN), an FC, and two residual connections for each LN. After the last TB, the attention representation is flattened and fed to a multi-layered perceptron (MLP) that consists of an FC followed by a classification layer with softmax activation. The architecture of the LT module is shown in Figure 4.

Overall, the input to the LT module consists of $N_{S}$ deep features generated by the ST module as described previously. Then the LT module learns the sequence representation of the deep features and generates the gesture label $g_{i}$ at the *clip level*.

### 2.5. Implementation Details

The training procedure was performed separately for the ST and LT modules. Initially, the decoder of the ST Model 1 and the FC of the ST Model 2 were trained for 15 epochs (warm-up training). The entire networks were then fine-tuned for 30 epochs. The employed optimizer was Adam with a learning rate of $10^{-4}$ for the warm-up phase and $10^{-5}$ during the fine-tuning phase. The loss function was the mean squared error for ST Model 1 and categorical cross entropy for ST Model 2. The LT module was trained using the Adam optimizer with a learning rate of $10^{-5}$ for 30 epochs and categorical cross-entropy as the loss function. The batch size was set to 10 throughout training due to computational memory constraints. All models were implemented in TensorFlow and trained on an Nvidia GTX 1060 GPU.

## 3. Results

### 3.1. Experimental Protocol

Three main models were evaluated: C3Dargmx, SSC3DTrans, and C3DTrans. All models take as input a video clip and output a gesture label at the *clip level*; no information about previous/forthcoming video frames is employed.

– C3Dargmx employs an argmax operation on the predictions generated by the ST Model 2. In particular, the gesture probabilities of the clip volumes are summed and the label with the highest probability is assigned to the clip. So, this model is trained for gesture prediction only at the volume level.
– C3DTrans employs the ST Model 2 and the LT model. The deep features from the clip volumes, generated by ST Model 2, are used as input to the LT Model, which in turn predicts the clip gesture. This model is trained for gesture prediction at the volume and clip levels.
– SSC3DTrans employs the ST Model 1 and LT model. This model works similarly with C3DTrans but now the LT model is fed with the embedding representations generated by the ST Model 1. Hence, this model is trained for gesture prediction only at the clip level, using the self-supervised features.

In addition, we employed two different sizes of annotated data for training (100% and 60%), so the total number of model variants under evaluation was six. For 100% annotation, all relevant models exploit the full annotated dataset. The option of 60% annotation was decided in order to examine the relative benefit of self-supervised learning on the whole training dataset in the case where only a fraction is annotated. To emulate this situation, the ST module of SSC3DTrans was trained on 100% of the training dataset, whereas the LT module (transformer) was trained on 60%. In contrast, C3Dargmx and C3DTrans were trained on 60% of the training dataset, given that both employ standard supervised learning. The main features of each model variant, with respect to the ST/LT modules and the size of annotated data, are presented in Table 3.

The performance of each model was evaluated separately for each surgical task (PT and KT). The videos were randomly split into five folds; every fold included clips and gesture instances from different trials. Using five-fold cross-validation, one of the five folds served as the test set (20%) and the other four folds served for training (80%). Another approach would be to employ ‘leave-one-out’ validation, wherein each run the video of a single trial is left out for testing. The latter though would require separate training of 40 models, which was very time-consuming for the low-end GPU employed (each of the 6 examined models required about 1 day of module-to-module training).

The models were tested for two experimental tasks. The first task focused on clip-level evaluation where the goal was to predict the correct gesture that a particular clip originates from. This is a particularly challenging problem considering that no information about the previous (or forthcoming) video frames is considered. The second task considered gesture-level evaluation. The goal was to predict the correct label of the whole gesture instance, which was simply achieved via argmax of the clips’ class probabilities. To obtain a dense representation of the entire gesture, its clips were processed sequentially with 80% overlap. For both experimental tasks, the performance was evaluated via accuracy (Acc) and F1 score. All the results are presented as mean values across the five-fold.

### 3.2. Experimental Results

In this section, we present the performance of the three proposed models trained on 100% and 60% of the training data, as well as the performance of two additional baseline models: I2D+LSTM-100 and I3D+LSTM-100, with the suffix 100 denoting that these models were trained on 100% of the annotated dataset. These models were based on fine-tuning the Inception2D (I2D) and Inception3D (I3D) using the frames and volumes of each clip, respectively. The feature vectors extracted from the second to last layer were fed to an LSTM network with 64 hidden units for clip classification. The LSTM module can be seen as an alternative to the LT model (transformer).

Table 4 shows the results of the *clip-level* evaluation with regard to the PT task. The lowest-performance network is I2D-LSTM-100 which is the only one employing a 2D CNN. The comparison of the models trained on 100% of the data shows that both C3DTrans and SSC3DTrans outperform I3D-LSTM by about 6% and 2%, respectively. Interestingly, C3Dargmx also outperforms I3D-LSTM, although the former lacks a time-varying model. By comparing the models trained on 100% vs. 60%, it can be seen that SSC3DTrans-60, which makes use of 100% for self-supervised training, proved to be the most robust with respect to the dataset size, showing only a marginal reduction of 0.3% in accuracy. In contrast, both C3DTrans and C3Dargmx showed a reduction of about 4% in accuracy.

From Table 5 it can be seen that the same pattern applies to the KT task as well. The proposed models outperformed both I2D-LSTM-100 and I2D-LSTM-100. Again, C3DTrans outperformed all other models both when using 100% and 60% of the dataset. Moreover, the self-supervised model (SSC3DTrans), has a performance similar to the best C3DTrans when using 60% of the dataset, something that was observed also for the PT task.

Next, we present the results of the second experimental task (*gesture-level* classification). For this task, we present only the results for C3DTrans and SSC3DTrans, which showed the most promising performance on clip-level evaluation. In addition, we present results for the two baseline models I2D+LSTM-100 and I3D+LSTM-100; again the suffix denotes that these models were trained on 100% of the dataset.

The results of the PT task shown in Table 6. As expected, I3D+LSTM outperformed I2D+LSTM by around 5% in accuracy and 4% in F1 score, denoting that the employment of 3D volumes provide a significant benefit over 2D frames. Comparing I3D+LSTM and C3DTrans, which both employ I3D as a backbone, shows that the transformer leads to a performance increase for both 100% and 60% of training data. Specifically, C3DTrans-100 showed about a 3–4% higher accuracy and F1-score compared to I3D+LSTM-100. A closer look (when training) on 60% of the data showed that the models experienced minor performance decreases compared to their counterparts trained on 100%. In addition, SSC3DTrans showed an accuracy decrement of only 0.2% while C3DTrans showed a 4% decrease. Hence, SSC3DTrans seems to be less vulnerable to a significant reduction in annotation (in our case from 100% to 60%).

The results of the KT task are shown in Table 7. The I3D+LSTM model outperformed I2D+LSTM by a close margin (0.6%). Similar to the PT task, the comparison between the I3D+LSTM model and C3DTrans show that the addition of a transformer offers a notable benefit (about a 3% increase in accuracy). The comparison between the models trained on 100% and 60% of the annotated data shows that SSC3DTrans experiences only a minor decrease in accuracy (1%) compared to that of C3DTrans (4.5%).

**Figure 5:**
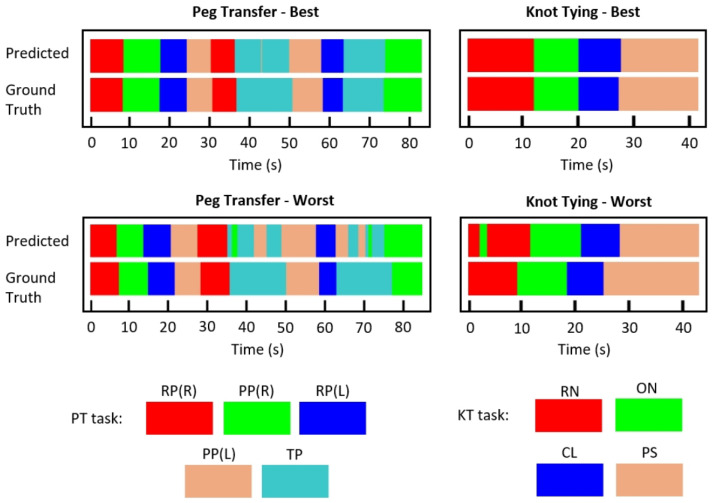
Color-coded ribbon visualization of the best and worst per-frame predictions, versus ground truth, for PT (left) and KT (right), using the C3DTrans-100 model.

Figure 5 presents the best and worst per-frame predictions using C3DTrans-100 for both surgical tasks, using color-coded ribbons. To obtain the predictions we extracted rolling clips with 1 frame step. The entire processing was performed online, meaning that the prediction for the n-th frame was based on frames [n-14,n]. Although trained for classification, rather than segmentation, the model was able to successfully identify the gesture transitions, indicating its potential for online prediction. It may be also noted that for the PT task, the model seems to slightly confuse PP and TP gestures.

**Figure 6:**
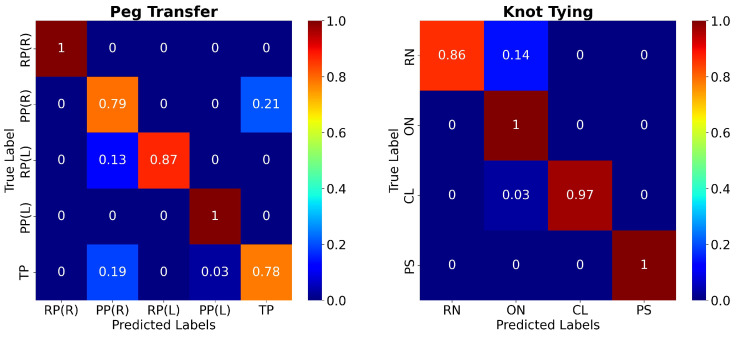
Color-coded confusion matrices of the proposed C3DTrans model for the PT (left) and KT (right) tasks. X- and Y-axes represent predicted and ground truth labels, respectively.

Figure 6 presents the confusion matrices for the two surgical tasks using the C3DTrans-100 model. For the PT task, it may be seen as a slight confusion between PP(R) and TP, which is in accordance with the color ribbons shown in Figure 5. Similarly, for the KT task there is a slight confusion between ON and RN. However, the network achieved 100% accuracy for the gestures RP(L), PP(L), (PT task), and ON, PS (KT task).

### 3.3. Evaluation of the JIGSAWS Dataset

In addition to our in-house dataset, we evaluated the performance of our best performing model (C3DTrans) on the JIGSAWS dataset, which includes three basic robotic tasks: suturing (SU), knot-tying (KT), and needle-passing (NP). The tasks are typically part of surgical skills training curricula. The dataset also provides two cross-validation schemes: leave-one-supertrial-out (LOSO) and leave-one-user-out (LOUO). In terms of data sources, tools kinematics, and video data from two video streams of the endoscopic camera are included in the dataset.

In this study, the input to our model was a single video stream, as compared to other techniques employing different images from the two available streams. The evaluation was performed with respect to the SU task using the LOUO scheme, given that most of the related work is evaluated with respect to this configuration.

Table 8 shows the accuracy comparison between our method and state-of-the-art techniques for surgical gesture recognition on the JIGSAWS dataset. The Table also includes some key features of the algorithms. Our method presents a similar or higher performance than the methods employing handcrafted features. The networks that outperformed our method use either additional data for training (CNN+LC-SC-CRF, 3D-CNN, MTL-VF) or the entire video stream as input (Symm dilation+attention). In contrast, our technique employs a single video stream with no additional training data, whereas the video input is processed in an online mode (rolling clip fashion).

## 4. Discussion

In this work, we present a video-based approach to online surgical gesture recognition based on supervised learning as well as self-supervised learning to cover the need for reducing the data annotation task. The supervised model is built upon a modular architecture that combines 3DCNN and transformer to capture short-term spatial features and long-term dependencies in the video stream, respectively. The self-supervised model is based on 3DCNN and next-frame prediction, aiming to learn spatiotemporal features that serve as input to the supervised model (transformer). The proposed models were evaluated on laparoscopic tasks using a custom-developed dataset, whereas prior art mainly focuses on robotic tasks where the tools and surgical maneuvers are inherently different.

The proposed model (C3DTrans) was able to classify fine-grained gestures from the surgical video of two laparoscopic training tasks. In particular, the accuracies of short video segments (clips) were 88% and 97.5% for the PT and KT tasks, respectively, whereas, for the entire gestures, it was >97.5% for both surgical tasks. As can be seen from Table 4, Table 5, Table 6 and Table 7, all model variants showed better performance for the KT task compared to the PT task. This may be due to the greater similarity of the PT gestures as compared to the KT ones. For example, the RP and PP gestures involve similar tool maneuvers handling the same object (peg). Moreover, these gestures are performed twice and sequentially during the task, implying a greater risk of misclassification. In contrast, the KT task requires a sequence of discrete gestures (RN→ON→CL→PS) handling different objects (needle and suture).

In addition, Figure 5 and Figure 6 provide a more detailed presentation concerning which gestures our model tends to misclassify. Regarding the PT task, the per-frame prediction and confusion matrix show that the model tends to confuse the TP gesture with the PP(L) and PP(R) gestures. These three gestures present similar movement patterns, as all three gestures start with the grasper holding the peg and end with the grasper placing the peg, either on the pegboard or on the other grasper. Regarding the KT task, we notice that the model confuses the RN with the ON gestures. Similar movement patterns may occur during those two gestures.

The use of unlabeled data for the self-supervising pretraining scheme showed promising results. In particular, the SSC3DTrans model variant proved more robust compared with its non-self-supervised counterparts, with a marginal decrease in performance. The comparison of the self-supervised versus the non-self-supervised counterparts in Table 4, Table 5, Table 6 and Table 7 reveals the following pattern. The self-supervised model variants performed worse when trained on 100% of the data when trained on 60% of the data the performance difference is reduced or even reversed. The initial difference might be attributed to the fact that the non-supervised short-term model is better at capturing latent features since it was trained for the same task as the complete network, as opposed to the self-supervised model. The reduction in performance indicates that this benefit diminishes with the reduction of labeled data.

With regard to the evaluation of the JIGSAWS dataset, we achieved a similar or better performance with other methods in the field. Table 8 provides an overview of these methods. It is worth mentioning that certain methods that outperform ours make use of additional data or employ future frames. For example, the CNN+LC–SC–CRF [29] uses the sensor values provided in the JIGSAWS dataset during the training procedure or while Symm dilation+attention [31] employs future frames.

The proposed method has some limitations that are worth mentioning. First, due to hardware constraints, the network was not trained end-to-end. As a result, the network was trained in a two-step manner, possibly limiting the network’s performance. Second, our method, such as all deep learning methods, requires a significant amount of labeled data in order to be trained, a process that is costly and time-consuming.

In conclusion, our proposed model achieved state-of-the-art accuracy on our in-house dataset while the proposed self-supervised pretraining scheme (next-frame prediction) opens the possibility for using much larger and partly annotated datasets, especially in minimally invasive surgery tasks where the camera provides an inherent data source. In addition, the application on the JIGSAWS dataset showed promising results. Future work may include the application of our framework on data coming from open surgery.

## Figures and Tables

**Table 1:** Gesture description.

Gesture Prefix	Task	Description
RP(R)	PT	Reach for the peg with the right grasper.
RP(L)	PT	Reach for the peg with the left grasper.
TP	PT	Transfer the peg between the graspers.
PP(R)	PT	Place the peg into the pegboard with the right grasper.
PP(L)	PT	Place the peg into the pegboard with the left grasper.
RN	KT	Reach for the needle.
ON	KT	Orient the needle.
CL	KT	Making the C-loop around the right grasper.
PS	KT	Reach for the suture end with the right grasper and pull the suture to lock the knot.

**Table 2:** Overview of the surgical gesture dataset.

Gestures:	RL	RR	PL	PR	T	RN	ON	CL	PS
#Instances	91	86	87	83	87	50	46	44	40
#Clips	530	393	888	1126	1586	271	342	462	708
Mean duration (s)	6.3	5.4	8.51	9.9	12.9	6.4	7.2	8.7	15.6
Min duration (s)	3.2	2.8	5.3	6.4	7.8	3.2	3.9	5.3	7.9
Max duration (s)	10.6	10.3	11.7	22.6	23.2	13.7	10.1	14.1	24.4

**Table 3:** Overview of the examined model variants with respect to the percentage of annotated data utilized for training.

Model Variant	% of Available Annotation in the Training Set	Is the Annotated Data Used for ST Training?	Is the Annotated Data Used for LT Training?
C3Dargmx-100	100	✓ (Model 2, trained on 100%)	– (argmax, no training)
SSC3DTrans-100	100	– (Model 1, self-supervised on 100%)	✓ (Transformer, trained on 100%)
C3DTrans-100	100	✓ (Model 2, trained on 100%)	✓ (Transformer, trained on 100%)
C3Dargmx-60	60	✓ (Model 2, trained on 60%)	– (argmax, no training)
SSC3DTrans-60	60	– (Model 1, self-supervised on 100%)	✓ (Transformer, trained on 60%)
C3DTrans-60	60	✓ (Model 2, trained on 60%)	✓ (Transformer, trained on 60%)

**Table 4:** Clip-level results of the peg transfer (PT) task. The best results column-wise are shown in bold.

Model Variant	% Acc	% F1
I2D+LSTM-100	81.1	67.2
I3D+LSTM-100	82.1	71.3
C3Dargmx-100	85.8	85.5
SSC3DTrans-100	84.1	81.3
C3DTrans-100	**88.0**	**87.2**
C3Dargmx-60	82.0	82.4
SSC3DTrans-60	83.8	81.3
C3DTrans-60	84.1	81.9

**Table 5:** Clip-level results of the knot-tying (KT) task. The best results column-wise are shown in bold.

Model Variant	% Acc	% F1
I2D+LSTM-100	87.0	85.1
I3D+LSTM-100	93.0	91.3
C3Dargmx-100	94.8	84.3
SSC3DTrans-100	93.2	91.4
C3DTrans-100	**95.2**	**95.2**
C3Dargmx-60	88.6	85.3
SSC3DTrans-60	92.4	91.1
C3DTrans-60	92.8	91.2

**Table 6:** Gesture-level results of the PT task. The best results column-wise are shown in bold.

Model Variant	% Acc	% F1
I2D+LSTM-100	89.8	88.6
I3D+LSTM-100	94.5	92.1
SSC3DTrans-100	92.0	90.2
C3DTrans-100	**97.5**	**96.2**
SSC3DTrans-60	91.8	90.1
C3DTrans-60	93.5	92.1

**Table 7:** Gesture-level results of the KT task. The best results column-wise are shown in bold.

Model Variant	% Acc	% F1
I2D+LSTM-100	94.6	94.1
I3D+LSTM-100	95.2	94.9
SSC3DTrans-100	96.3	95.4
C3DTrans-100	**97.9**	**96.2**
SSC3DTrans-60	95.5	93.1
C3DTrans-60	93.3	92.1

**Table 8:** Accuracy comparison for surgical gesture recognition on the SU task of JIGSAWS. The table also illustrates some key features of the methods.

Model	% Acc	#Cameras Employed	Difference Images	Trained on Additional Dataset	Applicable Online
CRF (dense) [5]	68.8	1	-	-	✓
MsM-CRF (STIP–STIP) [5]	66.3	1	-	-	✓
MsM-CRF (dense–dense) [5]	71.8	1	-	-	✓
CNN+LC-SC-CRF [29]	76.6	1	-	✓(Sensor Values)	✓
ST-GCN [30]	67.9	1	-	-	✓
MTL-VF [13]	82.1	1	-	✓	✓
3D-CNN [12]	84.0	1	-	✓(Kinetics)	✓
Symm dilation+attention [31]	90.1	1	✓	-	-
C3DTrans (Proposed)	75.8	1	-	-	✓

## Data Availability

Data are available upon reasonable request from the corresponding author.

## Abbreviations

The following abbreviations are used in this manuscript:

PT	peg transfer
KT	knot tying
JIGSAWS	JHU-ISI gesture skill assessment working set
JHU	Johns Hopkins University
ISI	Intuitive Surgical, Inc. (Sunnyvale, CA, USA)
DL	deep learning
HMM	hidden Markov model
CNN	convolutional neural network
TCN	temporal convolutional network
RS	robotic surgery
LS	laparoscopic surgery
LT	long-term
ST	short-term
I3D	Inception3D
LSTM	long short-term memory
TransConv2D	transpose 2D convolution
BN	batch normalization
TB	transformer block
SU	suturing
NP	needle-passing
LOSO	leave-one-supertrial-out
LOUO	leave-one-user-out
